# Patient-reported outcomes measures of X-linked hypophosphataemia participants: findings from a prospective cohort study in the UK

**DOI:** 10.1186/s13023-023-02620-w

**Published:** 2023-02-08

**Authors:** Sophie Cole, Maria T. Sanchez-Santos, Spyros Kolovos, Muhammad Kassim Javaid, Rafael Pinedo-Villanueva

**Affiliations:** 1 Nuffield Department of Orthopaedics, Rheumatology and Musculoskeletal Sciences, Nuffield Orthopaedic Centre, University of Oxford, Windmill Road, Oxford, OX3 7LD UK; 2 IQVIA, Athens, Greece

**Keywords:** X-linked hypophosphataemia, Rare diseases, Patient-reported outcome measures, Rickets

## Abstract

**Background:**

X-linked hypophosphataemia (XLH) is a rare genetic condition passed on through the X chromosome which causes multiple symptoms including weakened teeth, bones, and muscles. Due to the rarity of the condition, little is known about the health outcomes as reported by people with the disease. The objectives of this study were threefold: to characterise key patient reported outcome measures (PROMs) in adults with XLH, to identify clusters of symptom-severity groups based on PROMs, and to analyse the longitudinal progression of available PROMs.

**Methods:**

Data from 48 participants from the Rare and Undiagnosed Diseases cohort Study (RUDY) was used to analyse both cross-sectional and longitudinal patient-reported outcomes. We analysed data for health-related quality of life (HRQL): EuroQol 5 dimensions-5 levels (EQ-5D-5L), Short-form 36 (SF-36) Physical Component Score (PCS), and SF-36 Mental Component Score (MCS), sleep: Pittsburgh sleep quality index (PSQI) and Epworth Sleepiness scale (ESS), fatigue: Fatigue Severity Scale (FSS) and Functional assessment of chronic illness therapy-fatigue (FACIT-F), pain: Short form McGill pain questionnaire version 2 (SF-MPQ-2) and PainDETECT, and mental well-being: Hospital anxiety and depression scale (HADS) anxiety and depression. Summary statistics, tests of mean differences, mixed-effects models, and cluster analysis were used to describe and examine the various health dimensions of individuals with XLH.

**Results:**

Overall mean scores were EQ-5D-5L = 0.65, SF-36-PCS = 32.7, and SF-36-MCS = 48.4 for HRQL, ESS = 5.9 and PSQI = 8.9 for sleep, FSS = 32.8 and FACIT-F = 104.4 for fatigue, SF-MPQ-2 = 1.9 for pain, and HADS-depression = 4.7 and HADS-anxiety = 6.2 for mental well-being. 7% reported neuropathic pain (PainDETECT). Whilst many adults with XLH reported good outcomes, extreme or severe problems were reported across all outcomes. Cluster analysis identified that adults with XLH could be divided into two distinct groups, one reporting worse (35.3%) and the other better outcomes (64.7%) (less pain, fatigue, depression, and higher levels of sleep). Longitudinal analysis showed that FACIT-F and HADS-anxiety scores worsened slightly over two years with statistically significant (*p* < 0.05) time coefficients (b =  − 2.135 and b = 0.314, respectively).

**Conclusion:**

Although about two thirds of adult participants of the RUDY cohort with XLH report good health outcomes, for a considerable third much worse outcomes are reported. More research is needed to examine why some experience good and others poor health outcomes and the characteristics which identify them.

## Background

X-linked Hypophosphataemia (XLH) is a rare genetic condition of the X chromosome which causes low levels of phosphate in the bloodstream which in turn can lead to weakened bones, teeth, and muscles as well as other complications [1]. XLH is often diagnosed during childhood [1]. As the condition is X-linked dominant it is more prevalent among women. Due to the rarity of the condition, there is much which is not yet known. A systematic literature review found that for adults with XLH there is an ongoing burden and more research is need to improve awareness and understanding of the condition [2].

From previous research it has been shown that, compared to the general population, individuals with XLH experience greater pain, stiffness, reduced physical functional ability, and a higher rate of mortality compared to controls [3, 4]. It is not yet known the extent to which other health dimensions such as fatigue and depression impact the health-related quality of life (HRQL) of individuals with XLH. There is also no published evidence about whether the condition affects some health dimensions more than others or whether the size of these effects change with ageing. Although having a chronic illness can heavily impact an individual’s mental health [5, 6], the impact on the mental well-being of individuals with XLH has not been fully assessed. A clear characterisation and understanding of how XLH affects people with it would help guide future research and potential new treatment pathways.

The aim of this study was to describe the impact of XLH on a range of health dimensions for adults who have the disease. Three objectives were set to achieve this aim. The first objective was to cross-sectionally characterise a range of patient-reported outcome measures (PROMs) including sleep, pain, anxiety, and depression, and to measure their correlations. The second objective was to examine whether individuals with XLH can be clustered into different symptom-severity groups based upon their PROMs. The final objective was to describe the progression of the selected PROMs longitudinally.

## Methods

### Data

Data were extracted from questionnaires completed by adults with XLH participating in the Rare and Undiagnosed Diseases cohort Study (RUDY) from July 2014 to August 2019. The RUDY study is a prospective cohort of individuals with rare conditions launched in 2014 which collects data using a combination health surveys on patient characteristics (e.g. age, sex, and diagnosis) and PROM instruments [7]. Participants are self-selected into the study and the study was disseminated to member of the XLH-UK patient group. They are then invited to complete a series of different questionnaires every six months. Participants are free to complete any number of these questionnaires for a given a follow-up schedule and consequently not all would have been completed every 6 months.

Questionnaires were available for nine PROMs instruments measuring HRQL (EuroQol-5D-5L (EQ-5D-5L) and Short-form 36 (SF-36)), sleep (Pittsburgh sleep quality index (PSQI) and Epworth Sleepiness scale (ESS)), fatigue (Functional assessment of chronic illness therapy (FACIT)-F and Fatigue Severity Scale (FSS)), pain (Short form McGill pain questionnaire version 2 (SF-MPQ-2) and painDETECT), anxiety (Hospital anxiety and depression scale (HADS)) and depression (HADS). The ESS and FSS have a lower number of submitted questionnaires due to these being introduced into the RUDY database later than the rest. All instrument rating scales are fully outlined in Table 6 in the “Appendix”.

### Instruments

#### Health-related quality of life

EQ-5D-5L is an instrument designed to measure the HRQL of an individual through five dimensions: mobility, self-care, usual activities, pain or discomfort, and anxiety or depression [8]. For each dimension individuals can select one of five levels which range from no problems to extreme problems. The five responses of the individual can then be mapped to an English valuation set which assigns a score to represent their HRQL [9]. EQ-5D-3L index scores were also considered by mapping the EQ-5D-5L to the EQ-5D-3L using the crosswalk method [10]. EQ-5D-5L (3L) index scores can range between − 0.285 (− 0.594) and 1, anchored at 0 for death and with negative values representing states worse than death and 1 representing perfect health.

SF-36 also measures the HRQL of individuals [11]. The instrument has 36 questions which can be split into two components, physical health measured by the Physical Component Score (PCS) and mental health by the Mental Component Score (MCS). Participants of the RUDY Study completed the first US version SF-36 of the instrument and consequently results from the study were normalised using US population norms [12]. The PCS and MCS can range between 0 and 100.

#### Sleep

PSQI is an instrument used to measure an individual’s quality of sleep [13]. It has seven components, and overall scores can range from 0 to 21. The higher the score, the poorer the quality of sleep. Those who report a score greater than five can be categorised as ‘poor sleepers’ whilst those who score five or under can be categorised as ‘good sleepers’ [13].

ESS is an eight-item instrument which evaluates the sleepiness of an individual into five separate categories: lower normal, higher normal, mild excessive, moderate excessive, and severe excessive [14]. Questions are answered with a four-point Likert scale from ‘would never doze’ to a ‘high chance of dozing’. Scores range from 0 to 24. Those who report a score from 0 to 5 are categorised as lower normal daytime sleepiness, 6–10 is categorised as higher normal daytime sleepiness, 11–12 as mild excessive daytime sleepiness, 13–15 is considered moderate excessive daytime sleepiness, and 16–24 is categorised as severe excessive daytime sleepiness.

#### Fatigue

FACIT-F was designed to measure the level of fatigue of an individual [15]. Originally designed to measure HRQL of cancer patients, the instrument was adapted to make it applicable for a broader range of patients. The FACIT-F can be split into five different subscales: physical well-being (PWB), social well-being (SWB), emotional well-being (EWB), functional well-being (FWB), and fatigue subscale (FS). The lower the score, the greater the fatigue.

FSS is an instrument designed to measure fatigue [16]. It contains nine statements which can be graded with a seven-point Likert scale ranging from strongly disagree to strongly agree. The higher the score, the greater the fatigue.

#### Pain

SF-MPQ-2 is an instrument designed to measure whether an individual has neuropathic pain [17, 18]. Individuals are asked to scale 22 questions regarding four pain components (continuous pain, intermittent pain, neuropathic pain, and affective descriptors) between zero and 10. The total score is given by calculating the mean score of the 22 items with a higher score represents a greater level of pain experienced.

PainDETECT measures neuropathic pain [19] as well. Scores range between − 1 and 38, those who score 12 or less are unlikely to have neuropathic pain, those who score 19 or more are said to have a neuropathic pain component, and for those who score between 12 and 19 it is categorised as unclear. The proportion with neuropathic pain was used as the primary outcome for this score.

#### Mental well-being

Hospital anxiety and depression scale (HADS) is an instrument which measures both anxiety and depression [20]. The instrument is made up of 14 questions which are split into two subsections: one measuring anxiety and the other depression. Individual’s responses to each question can range between zero and three. The two parts are then added up separately. Each total score can range between 0 and 21, with those whose results are between eight and 11 being considered ‘mild’ whilst individuals who report a total score greater than 11 are categorised as having either anxiety or depression, respectively.

### Analysis

#### Cross-sectional

Summary statistics of the sample demographics (including age and sex) were calculated to verify whether the extracted data from RUDY was representative of the population of individuals with XLH. Mean scores were calculated for the first of each of the nine submitted questionnaires and tests undertaken for statistical differences of age and sex using the T-test and ANOVA, respectively. Based on clinical expert advice (MKJ), age was split into three groups: < 40 years, 40–60 years, and > 60 years old. Where appropriate, overall instrument scores were broken down into their instrument categories and further compared across the age groups. The EQ-5D-5L instrument was broken down by its dimensions for the overall sample and split by sex. To investigate whether the health dimensions of each participant were correlated with each other, repeated-measures correlations [21, 22] were calculated between all pairs of questionnaires for the same individuals. All paired submissions were used. This analysis allowed to identify the relationship (if any) of each health instrument with one another.

#### Clustering

Cluster analysis using the k-means method was applied to analyse whether participants could be grouped into clusters of distinct levels of severity according to questionnaires’ scores. The elbow and average silhouette method were implemented to identify a suitable number of clusters for the analysis. From the elbow plot, the optimal number of clusters is the point where the sum of squares starts to level off, whilst for the average silhouette method it is the point at which the average silhouette width is maximised. To generate the clusters, the first submission scores of the EQ-5D-5L, SF-36, FACIT-F, PainDETECT, SF-MPQ-2, PSQI, and HADS were used. FSS and ESS were excluded from the analysis due to an insufficient number of first submissions. Due to the questionnaires’ scores range variability, mean scores were standardised to ensure that no instrument had a greater weighting on the cluster analysis than others. The natural mean instrument scores were then reported for each cluster. A sensitivity analysis was undertaken by removing instruments with low response rates in the first submissions to allow for a larger sample size and better generalisability. Results from the sensitivity analysis were then compared to the original cluster analysis.

#### Longitudinal

Mixed-effects regression models were run to investigate whether subsequent scores reported by the participants changed over time. The first four questionnaire submissions of each instrument by participants were used. Age and sex were used as controls.

All analyses were completed using statistical software R [23] and additional relevant packages [24–42].

## Results

**Fig. 1:**
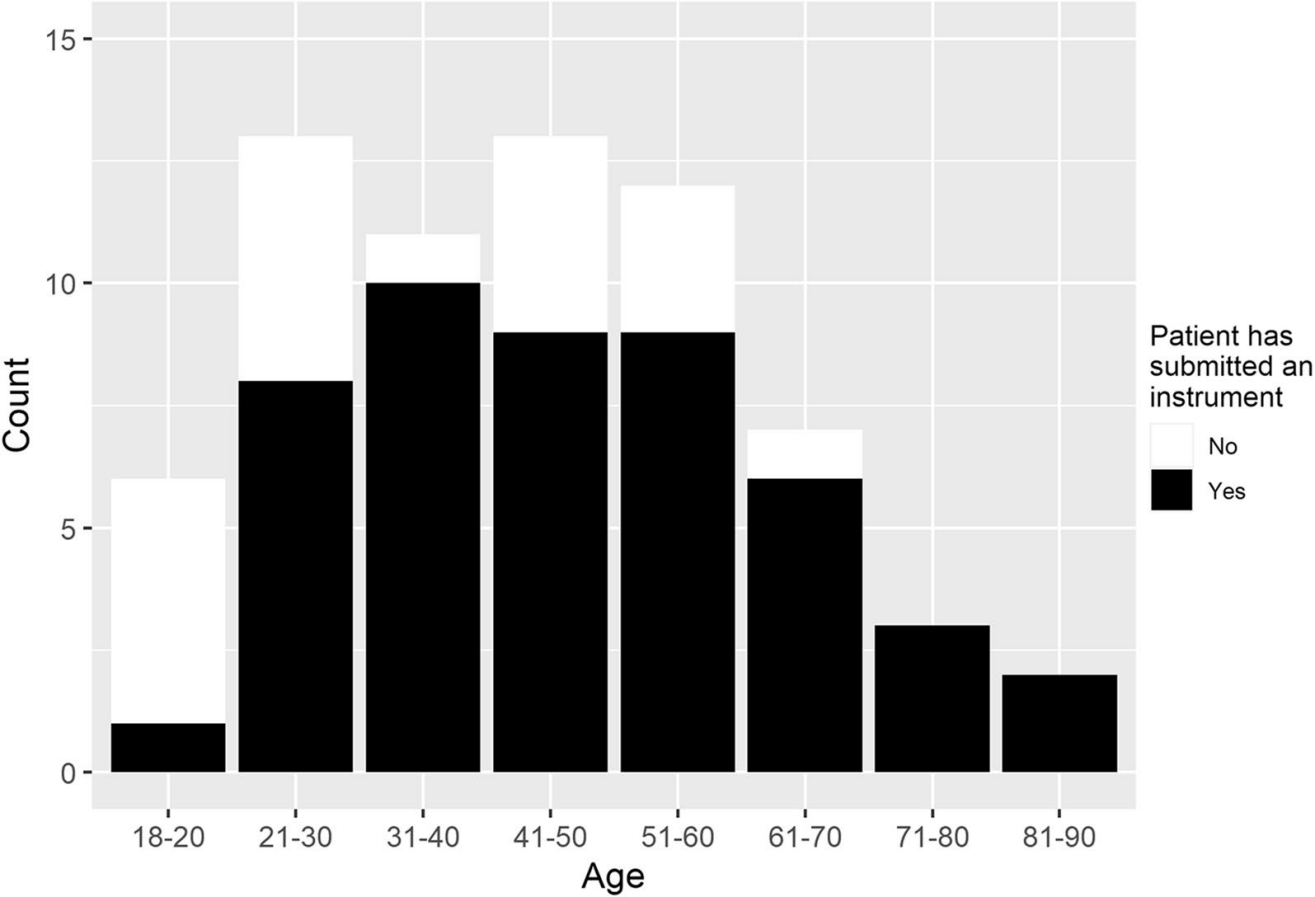
A comparison of those registered in the RUDY study and those who have submitted an instrument by age

The sample constituted of 48 participants who submitted at least one of the nine questionnaires, the majority of whom were female (77.1%). Questionnaires were completed by participants aged between 28 and 85, with most registered study participants submitting at least one PROMs instrument except for those under 21 where only one of the six participants did (see Fig. 1). Median age was 46 years, with the highest number of submissions made by those aged 31–40 years old.

The EQ-5D-5L had the highest number of submitted questionnaires, whilst ESS and FSS had the fewest submissions by participants (see Fig. 4 in the “Appendix”).

### Cross-sectional analysis

**Fig. 2:**
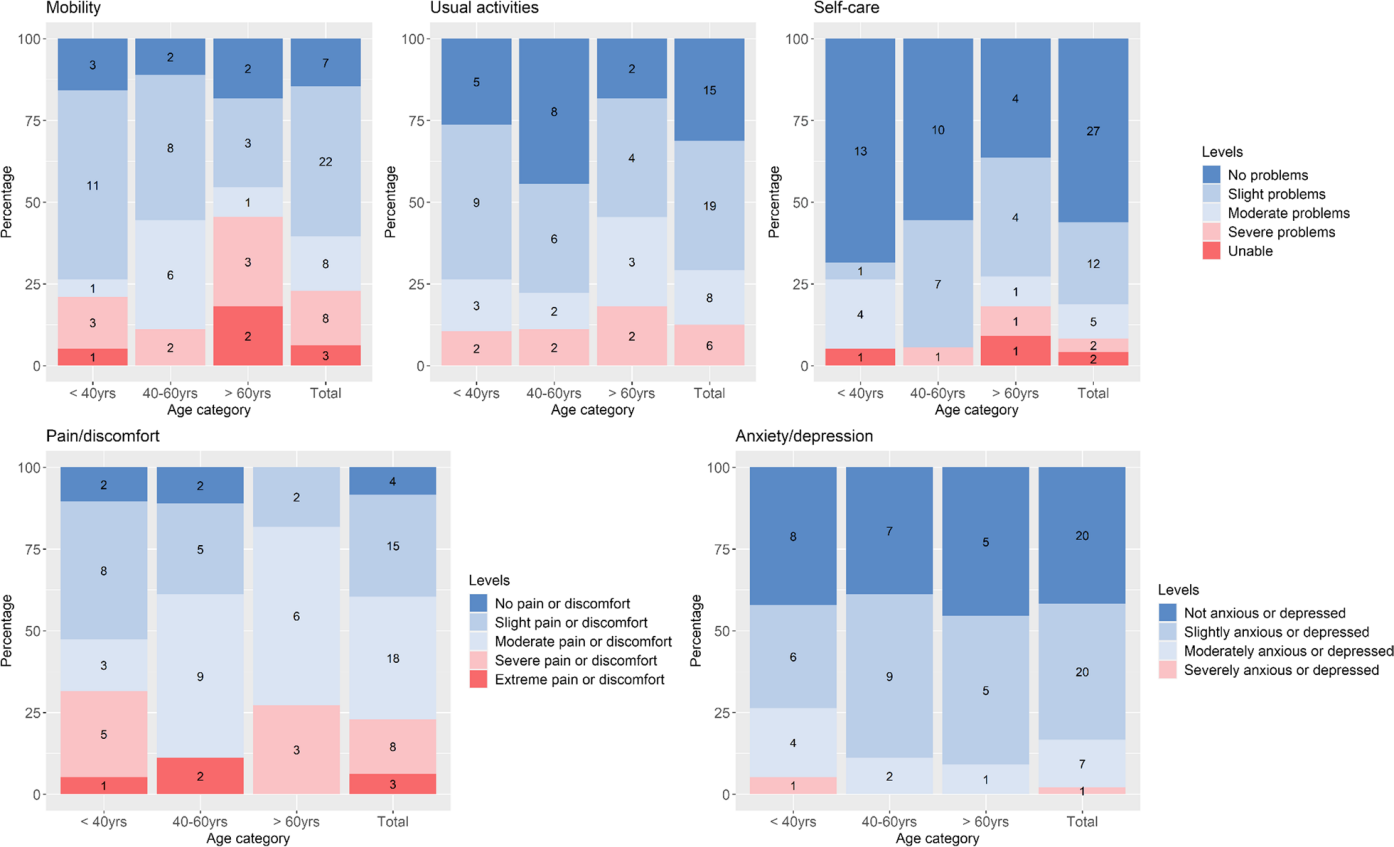
EQ-5D-5L: dimension levels by age group (count of individuals given within the figure)

Mean scores per instrument for the whole sample as well as for men and women separately are shown in Table 1. Scores by age group are shown in Fig. 2 and Table 7 in the “Appendix”. Severe or extreme problems were identified across many questionnaires, particularly in the EQ-5D-5L dimensions mobility (23%) and pain (23%) (see Fig. 2). Furthermore, low mean scores were reported for the FACIT-F components for functional wellbeing (mean = 18.2) and fatigue (mean = 31.3) (see Tables 7, 8, 9, 10 in the “Appendix”). No statistically significant difference in mean scores by age group or by sex was found except for the sleep instrument, PSQI, which found that males (mean = 6.4) experience slightly better quality of sleep than females (mean = 9.6) (Table 1).

**Table 1:** Cross-sectional summary statistics, by sex

Overall instrument score	Total					Female				Male				
	n	Mean	SD	Median	Range of submission dates	n	Mean	SD	Median	n	Mean	SD	Median	T-test *P*-value
*Health related quality of life*														
EQ-5D-5L Index score	48	0.651	0.270	0.734	07/2014–08/2019	37	0.645	0.261	0.730	11	0.669	0.312	0.758	0.826
EQ5D VAS score	48	62.48	25.74	70.00	07/2014–08/2019	37	61.32	25.74	70.00	11	66.36	26.57	70.00	0.586
EQ5D-3L Index score (crosswalk)	48	0.554	0.300	0.654	07/2014–08/2019	37	0.550	0.285	0.654	11	0.567	0.361	0.642	0.887
SF-36- physical component score	44	32.67	14.19	31.66	07/2014–08/2019	35	31.31	11.96	31.19	9	37.98	20.82	36.27	0.379
SF-36- mental component score	44	48.38	10.41	49.28	07/2014–08/2019	35	48.50	11.19	50.28	9	47.89	7.14	47.82	0.843
*Fatigue*														
FSS score	23	32.83	14.04	30.00	09/2018–08/2019	20	33.40	13.22	31.50	3	29.00	21.93	20.00	0.764
FACIT-F score	43	104.40	29.31	108.00	04/2015–08/2019	35	102.70	30.01	106.00	8	111.90	26.47	115.00	0.404
FACIT-F- physical well-being	43	18.52	6.30	20.00	04/2015–08/2019	35	18.49	6.31	20.00	8	18.75	7.11	20.50	0.921
FACIT-F- social well-being	43	20.59	5.15	22.00	04/2015–08/2019	35	20.30	5.47	21.00	8	21.65	3.97	22.58	0.451
FACIT-F- emotional well-being	43	15.86	4.67	16.00	04/2015–08/2019	35	15.63	4.74	15.00	8	16.50	4.78	17.00	0.685
FACIT-F- functional well-being	43	18.25	5.49	18.00	04/2015–08/2019	35	17.97	5.58	18.00	8	19.12	5.64	19.50	0.637
FACIT-F- fatigue subscale	43	31.33	13.28	33.00	04/2015–08/2019	35	30.29	13.37	33.00	8	35.88	12.69	38.00	0.290
*Pain*														
SF-MPQ-2 score	46	1.94	1.51	1.77	07/2014–08/2019	37	2.05	1.52	1.86	9	1.46	1.47	0.73	0.304
painDETECT score	43	9.28	6.25	9.00	07–2014-08/2019	36	9.39	6.29	9.00	7	8.71	6.55	9.00	0.808
Negative, n (%)	28	65.12				23	63.89			5	71.43			
Unclear, n (%)	12	27.91				11	30.56			1	14.29			
Positive, n (%)	3	6.98				2	5.56			1	14.29			
*Sleep*														
ESS score	23	5.87	3.68	5.00	09/2018–08/2019	20	6.25	3.57	5.50	3	3.33	4.16	2.00	0.349
PSQI score	41	8.88	4.12	9.00	07/2014–06/2019	32	9.56	4.05	9.50	9	6.44	3.61	5.00	**0.043**
*Mental wellbeing*														
HADS Anxiety score	41	6.17	3.83	6.00	04/2015–06/2019	32	6.28	3.56	6.00	9	5.78	4.89	7.00	0.779
Normal, n (%)	26	63.41				21	65.62			5	55.56			
Mild, n (%)	10	24.39				8	25.00			2	22.22			
Moderate/severe, n (%)	5	12.20				3	9.38			2	22.22			
HADS Depression score	41	4.71	3.49	4.00	04/2015–06/2019	32	4.69	3.32	4.00	9	4.78	4.27	4.00	0.954
Normal, n (%)	35	85.37				28	87.50			7	77.78			
Mild, n (%)	2	4.88				1	3.12			1	11.11			
Moderate/severe, n (%)	4	9.76				3	9.38			1	11.11			

The sample is the first submitted instrument from each patient

EQ-5D-5L (3L), EuroQol 5 dimensions-5 levels (3 levels); VAS, Visual analogue scale; SF-36, Short-form 36; PCS, Physical Component Score; MCS, Mental Component Score; FSS, Fatigue Severity Scale; ESS, Epworth Sleepiness scale; PSQI, Pittsburgh sleep quality index; FACIT-F, Functional assessment of chronic illness therapy-fatigue; SF-MPQ-2, Short form McGill pain questionnaire version 2; HADS, Hospital anxiety and depression scale

Correlation between questionnaires was low to moderate (r < 0.6) with some of them being statistically significant (*p* < 0.05), not only within the same health dimension (both pain measures, McGill Pain and PainDETECT), but also across dimensions (FACIT-F fatigue was correlated with PSQI sleep score) (Table 2). The magnitude of the correlation coefficients ranged from r =  − 0.019 between SF-36-MCS and painDETECT, to r = 0.579 between PSQI and FSS. Of the statistically significant correlations, the highest correlation was between FACIT-F and SF-36-MCS with a coefficient of r = 0.513 (*p* < 0.001).

**Table 2:**
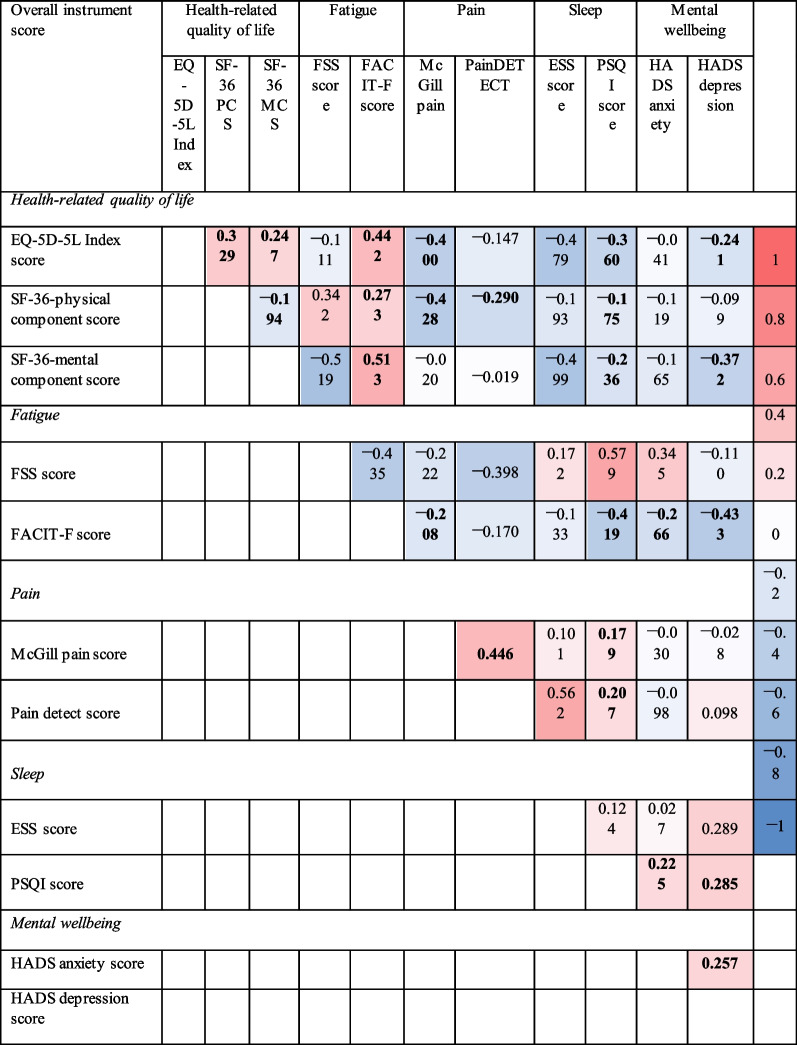
Repeated measures correlation test between scores (correlation, r)

Results in bold are statistically significant at a 5% level

EQ-5D-5L, EuroQol 5 dimensions-5 levels; SF-36, Short-form 36; PCS, Physical Component Score; MCS, Mental Component Score; FSS, Fatigue Severity Scale; ESS, Epworth Sleepiness scale; PSQI, Pittsburgh sleep quality index; FACIT-F, Functional assessment of chronic illness therapy-fatigue; SF-MPQ-2, Short form McGill pain questionnaire version 2; HADS, Hospital anxiety and depression scale

### Cluster analysis analysis

**Fig. 3:**
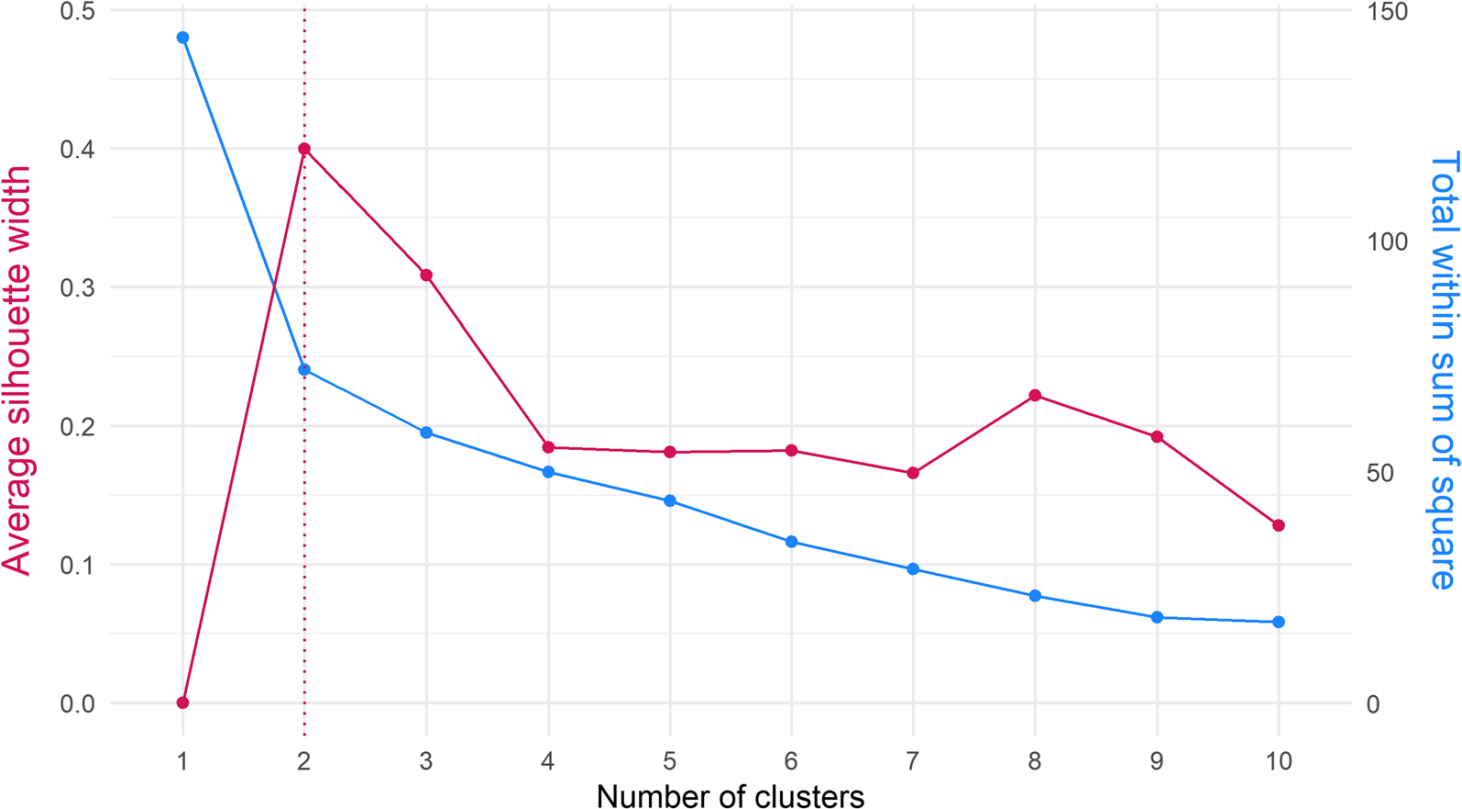
Cluster analysis—average silhouette and Elbow plot

**Fig. 4:**
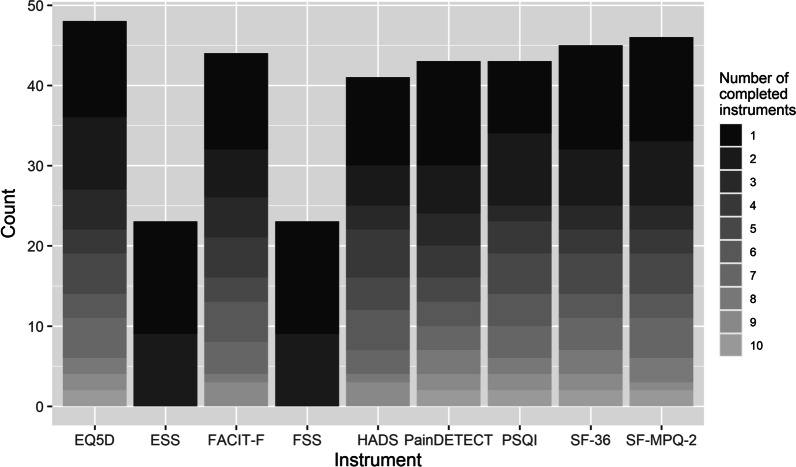
Stacked histogram of the number of instruments completed by the RUDY participants

**Fig. 5:**
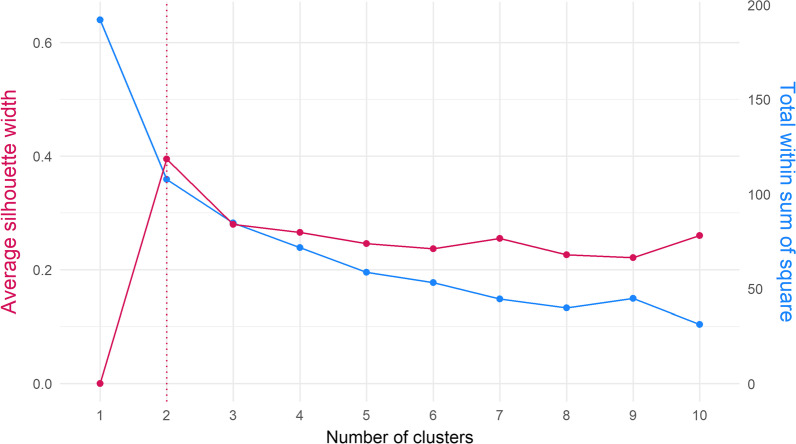
Cluster sensitivity analysis—average silhouette and Elbow plot

Of the 48 participants, 17 completed all seven PROMs (EQ-5D-5L, SF-36, FACIT-F, PainDETECT, SF-MPQ-2, PSQI, and HADS) at their first submission, with HADS, FACIT-F, and to a lesser extent PainDETECT, accounting for most of those dropped. Of this complete case subsample, 76.5% were female and the median age was 41 years, both slightly lower than the percentage of females (77%) and median age (46 years) of the main sample. The elbow and average silhouette analysis found that the optimal number of clusters was two (Fig. 3), as that is the point at which the sum of squares started to level off and the average silhouette width reached its maximum point. The 17 participants were then clustered into two groups, a “higher-score” group and a “lower-score” group (see Table 3). 11 participants were included in the “higher-score” group and reported higher mean HRQL scores, better quality of sleep, less fatigue, less pain, lower likelihood of being categorised as with neuropathic pain, and lower levels of depression. There was no significant difference in mean anxiety scores between the two groups (“higher-score” HADS = 6.73 vs. “lower-score” HADS = 6.50), although after categorisation there is a marked difference in the percentage of participants with mild anxiety (45.5% in the “higher score” group vs. 16.7% in the “lower score” group). For the sensitivity analysis, FACIT-F and HADS were removed as they reported the lowest response rate. This resulted in 33 participants who completed all five remaining PROMS at their first submission: EQ-5D-5L, SF-36, PainDETECT, SF-MPQ-2, and PSQI. The elbow and average silhouette analysis once more found that the optimal number of clusters was two (Fig. 5). Participants were equally clustered into a “higher-score” group and a “lower-score” group (see Table 11). Those in the higher-score group reported health outcomes of a better quality of life, less pain, better quality of sleep and less neuropathic pain. Mean scores differed slightly from the original cluster analysis.

**Table 3:** Cluster analysis, mean instrument scores

Instrument	Cluster 1 (n = 11) “Higher-score” (mean)	Cluster 2 (n = 6) “Lower-score” (mean)
EQ-5D-5L	0.817	0.288
PSQI	7.64	12.67
SF-36-PCS	45.40	19.54
SF-36-MCS	51.31	43.40
SF-MPQ-2	1.03	3.58
painDETECT	5.64	14.17
Negative, n (%)	10 (90.91)	2 (33.33)
Unclear, n (%)	1 (9.09)	3 (50.00)
Positive, n (%)	0 (0.00)	1 (16.67)
FACIT-F	125.32	78.00
HADS-anxiety	6.73	6.50
Normal, n (%)	5 (45.45)	4 (66.67)
Mild, n (%)	5 (45.45)	1 (16.67)
Moderate/severe, n (%)	1 (9.09)	1 (16.67)
HADS-depression	3.18	8.17
Normal, n (%)	11 (100%)	3 (50.00)
Mild, n (%)	0	1 (16.67)
Moderate/severe, n (%)	0	2 (33.33)

EQ-5D-5L, EuroQol 5 dimensions-5 levels; SF-36, Short-form 36; PCS, Physical Component Score; MCS, Mental Component Score; PSQI, Pittsburgh sleep quality index; FACIT-F, Functional assessment of chronic illness therapy-fatigue; SF-MPQ-2; Short form McGill pain questionnaire version 2; HADS, Hospital anxiety and depression scale

### Longitudinal analysis

All questionnaires were found to have fluctuating means and median scores across the four time points except for SF-MPQ-2 and PSQI which showed a slight improvement over time (approximately two years) (see Table 4). The mixed effects models showed that only FACIT-F and HADS-anxiety had statistically significant (*p* < 0.05) time coefficients (b =  − 2.135 and b = 0.314, respectively), both indicating that scores worsened slightly overtime (approximately two years) (see Table 5). Within the FACIT-F score, the only component with a statistically significant time coefficient was the functional well-being subscale (b =  − 0.754), proving hence to be the driver of change reported over time.

**Table 4:** Longitudinal summary statistics

Instrument	Issue 1				Issue 2				Issue 3				Issue 4			
	n	Mean	Median	SD	n	Mean	Median	SD	n	Mean	Median	SD	n	Mean	Median	SD
*Health related quality of life*																
EQ-5D-5L Index score (VT)	47	0.650	0.738	0.273	23	0.729	0.801	0.176	25	0.689	0.733	0.189	27	0.700	0.751	0.238
EQ5D VAS score	47	63.17	70	25.56	23	70.13	70	18.88	25	65.24	70	21.80	27	67.37	72	22.95
EQ5D-3L Index score (CW)	47	0.552	0.654	0.303	23	0.630	0.706	0.218	25	0.601	0.642	0.201	27	0.603	0.679	0.269
SF-36- physical component score	42	33.32	32.91	14.1	22	36.59	35.53	10.41	26	31.37	30.26	11.57	26	36.29	35.18	14.72
SF-36- mental component score	42	48.72	49.99	10.54	22	48.82	51.84	10.03	26	50.86	54.62	11.47	26	47.71	49.59	11.80
*Fatigue*																
FACIT-F score	24	105.26	109.5	29.10	25	108.93	106	26.81	26	105.62	112.5	28.50	27	106.38	106	27.00
FACIT-F- physical well-being	24	18.96	19.5	6.48	25	19.16	20	5.84	26	18.73	19.5	5.70	27	19.78	21	5.58
FACIT-F- social well-being	24	20.89	21.5	5.25	25	20.29	19.83	5.10	26	19.69	20.5	5.60	27	19.3	21	5.69
FACIT-F- emotional well-being	24	16.75	17	4.12	25	16.28	17	4.87	26	16.42	18	5.25	27	16.74	18	4.34
FACIT-F- functional well-being	24	18.42	18.5	5.51	25	18.80	19	5.39	26	17.38	17.5	5.42	27	17.44	18	5.56
FACIT-F- fatigue subscale	24	30.25	33.5	13.87	25	34.40	34	10.81	26	33.38	34.5	12.23	27	33.11	30	12.60
*Pain*																
McGill pain score	43	1.89	1.68	1.47	22	1.83	1.48	1.47	24	1.82	1.61	1.29	26	1.76	1.09	1.50
Pain detect score	40	9.33	9	6.32	21	10.90	10	8.32	24	10.38	8.5	7.72	25	9.56	9	6.06
*Sleep*																
PSQI score	41	8.88	9	4.12	23	8.48	8	4.05	24	8.42	8	4.21	26	7.92	8	3.55
*Mental wellbeing*																
HADS anxiety score	24	6.17	6	3.51	22	5.64	5	4.04	25	6.92	6	3.95	25	6.28	6	3.84
HADS depression score	24	4.58	4	3.13	22	4.45	4	3.43	25	4.92	4	3.12	25	4.56	4	2.71

The sample is all submitted questionnaires from the first four submissions

EQ-5D-5L (3L), EuroQol 5 dimensions-5 levels (3 levels); VAS, Visual analogue scale; SF-36, Short-form 36; PCS, Physical Component Score; MCS, Mental Component Score; FSS, Fatigue Severity Scale; ESS, Epworth Sleepiness scale; PSQI, Pittsburgh sleep quality index; FACIT-F, Functional assessment of chronic illness therapy-fatigue; SF-MPQ-2, Short form McGill pain questionnaire version 2; HADS, Hospital anxiety and depression scale

**Table 5:** Mixed-effects models (random intercept and fixed slope) controlling for age and sex

	Total sample				Total sample		
	Instrument (n)	Coefficient	*p*-value		Instrument (n)	Coefficient	*p*-value
*EQ-5D-5L Index score*				*FACIT-F score*			
Intercept	122	0.742	0.000	Intercept	102	107.985	0.000
Time	122	− 0.006	0.483	Time	102	**− 2.135**	**0.038**
*EQ5D VAS score*				*FACIT-F- physical well-being*			
Intercept	122	63.617	0.000	Intercept	102	19.894	0.000
Time	122	0.445	0.716	Time	102	− 0.038	0.861
*EQ5D-3L Index score*				*FACIT-F- social well-being*			
Intercept	122	0.688	0.000	Intercept	102	22.825	0.000
Time	122	− 0.003	0.699	Time	102	− 0.571	0.079
*SF-36- physical component score*				*FACIT-F- emotional well-being*			
Intercept	116	41.502	0.000	Intercept	102	14.693	0.000
Time	116	0.242	0.576	Time	102	0.132	0.567
*SF-36- mental component score*				*FACIT-F- functional well-being*			
Intercept	116	43.432	0.000	Intercept	102	22.175	0.000
Time	116	− 0.467	0.371	Time	102	**− 0.754**	**0.003**
*PSQI score*				*FACIT-F- fatigue subscale*			
Intercept	114	7.652	0.000	Intercept	102	28.556	0.000
Time	114	− 0.117	0.473	Time	102	− 0.729	0.136
*HADS anxiety score*				*McGill pain score*			
Intercept	96	5.346	0.004	Intercept	115	1.522	0.019
Time	96	**0.314**	**0.031**	Time	115	0.011	0.865
*HADS depression score*				*Pain detect score*			
Intercept	96	3.385	0.029	Intercept	110	4.977	0.095
Time	96	0.229	0.162	Time	110	0.202	0.464

Time coefficients in bold are statistically significant at a 5% level

EQ-5D-5L (3L), EuroQol 5 dimensions-5 levels (3 levels); VAS, Visual analogue scale; SF-36, Short-form 36; PCS, Physical Component Score; MCS, Mental Component Score; FSS, Fatigue Severity Scale; ESS, Epworth Sleepiness scale; PSQI, Pittsburgh sleep quality index; FACIT-F, Functional assessment of chronic illness therapy-fatigue; SF-MPQ-2, Short form McGill pain questionnaire version 2; HADS, Hospital anxiety and depression scale

## Discussion

We have characterised and analysed a range of health dimensions for adults with XLH, grouped the participants into two separate clusters, and found little change in outcomes over time. Many of the adults with XLH reported no or slight problems with mobility (EQ-5D-5L, 60.41%) and pain (EQ-5D-5L, 39.58%), normal levels of sleepiness (ESS, 91.30%), no neuropathic pain (painDETECT, 65.12%), and normal levels of anxiety (HADS, 63.41%) and depression (HADS, 85.37%). However, a distinct few reported having severe or unable difficulty with mobility (EQ-5D-5L, 22.92%) and pain (EQ-5D-5L, 22.92%), and low mean scores for fatigue (FACIT-F FS, 31.33) and functional well-being (FACIT-F FWB, 18.25). These results are supported by previous research which found that for adults with XLH, pain was the most prominent symptom which then additionally impacted physical functioning [43]. Furthermore, a greater weighting of females in the RUDY study is representative of the demographic of the XLH population and supports previous findings [44, 45].

The correlation between mental health and fatigue was found to be the highest of all the PROMs. Except for females experiencing worse quality sleep than males, no other statistically significant difference was found between males and females. This result is supported by previous research, which also found no significant difference in the symptom severity between males and females [46].

Cluster analysis found that, even with a small sample of individuals with XLH, they could be divided into two distinct groups, where one group reported better quality of life, fatigue, sleep, pain, and depression than the other group. For those who report good outcomes it may be that they have learnt to live with and manage condition or have a good support system of care. Sensitivity analysis increasing the sample size by removing instruments with the lowest response rates at first submission also identified two clusters with very similar PROMs scores. Further analysis of these group differences could in the future enable learnings to be found from those who report good outcomes and a better streamline of care and resources to those who experience the worst symptoms. Including participants’ genetic information may provide further insight into these group differences. However, this is not yet collected by the RUDY study and consequently could not be explored in this analysis. More research with a larger sample size is needed to confirm that these group differences persist.

Over a two-year period, we found evidence of stability across the health outcomes with signals of worsening anxiety and fatigue. If this deterioration were to continue beyond the two years, this could significantly impact the well-being of people with XLH suffering from these ailments. However, for all other health dimensions there was no evidence of change over the two years examined. We are not able to compare the FACIT-F fatigue subscale scores of our XLH sample against a UK-based general population study, but our findings report a much lower mean (31.33) than that of the German population obtained from a household survey via a nationwide random sampling (43.5) [47]. Although differences may be explained by both differences in health between adults with XLH and the general population and differences in the UK and German populations, it is likely that the former dominates suggesting that on average people with XLH experience a greater level of fatigue than the general population.

Mean HRQL (SF-36-PCS and SF-36-MCS) of individuals with XLH (SF-36-PCS = 32.67 and SF-36-MCS = 48.38) is comparable to scores reported by people with musculoskeletal pain (SF-36-PCS = 32.93 and SF-36-MCS = 48.03) [48]. However, our mean SF-36-PCS and SF-36-MCS results were lower (worse) than previous research of XLH populations has found [45]. This difference may be due to the reported use of phosphate and/or vitamin D supplements reported by the latter research. In addition, part of the differences could be due to differences in study design; RUDY is an online study where participants can complete instruments in their own time, whereas the previous research was a hospital-based study, where applicants would complete instruments on paper as part of the hospital visit. Both XLH study populations reported a lower mean SF-36-PCS than mean SF-36-MCS suggesting that the physical impact of the condition may be greater than the mental impact. We found mean HRQL of adults with XLH (EQ-5D-5L = 0.651) to be higher than that of a Spanish study of adults with XLH (EQ-5D-5L = 0.562) [49]. This difference may be due to limited generalisability between UK and Spanish populations or the latter study identifying their participants through specialized clinics which may infer a more severe case mix. Comparison of UK and Spanish EQ-5D population norms show that reported health-related quality of life in the UK is lower than in Spain (0.852 and 0.914, respectively) [50].

A comparison with previous research shows that adults with XLH participating in RUDY report less neuropathic pain on average than those diagnosed with musculoskeletal pain (mean = 9.28 vs. mean = 17.13) [48]. We also found that, although the mean anxiety score of both male and female adults with XLH participating in RUDY (M = 5.78 and F = 6.28) were similar to UK population norms (M = 5.51 and F = 6.78), the percentage of those with moderate/severe anxiety was lower in RUDY (F: 9.4% and M: 22.2% compared to F: 19.0% and M: 12.5%) [51]. This is consistent with previous studies reporting the resilience and acceptance of people living with some rare diseases [52, 53]. Conversely, the proportion of participants with moderate/severe depression was slightly higher for the RUDY sample than the UK population comparator (F: 9.4% and M: 11.1% vs. F: 6.9% and M: 6.9%) [51]. However, it should be noted that the RUDY study covered a slightly wider age range than the comparator study (25–65 years old) and that, additionally, differences between people with XLH and the general population, as well as across countries, could stem from varying and relatively lower levels of deprivation experienced by people with XLH [3].

This analysis benefited from the richness of the data collected through the RUDY study, with questionnaires measuring a wide range of health dimensions. This enabled the health dimensions to be compared and investigated simultaneously developing the characterisation of the HRQL of people with XLH. Furthermore, RUDY participants who complete an instrument multiple times enable longitudinal analysis of how their PROMs change overtime. This gives further insight into how the condition develops and changes overtime and a better understanding of the experiences of individuals with a diagnosis of XLH.

Our study faced limitations. Due to the rarity of the disease, the sample size is small which in turn may limit the power of our results. A small sample size also brings doubt as to whether the sample is truly representative of the XLH population. Furthermore, as participants are free to complete any number of questionnaires for a given submission this means that some questionnaires results are missing for a given submission and consequently limited the comparability of the questionnaires. Finally, as RUDY is self-reported we were unable to validate the accuracy of clinical diagnosis but the diagnostic accuracy in other rare bone disorders is high [54, 55].

## Conclusions

We have characterised important health aspects and identified a cluster of adults living with XLH who report worse outcomes across the range of musculoskeletal and non-musculoskeletal health dimensions. More research is needed to better identify the characteristics of those who consistently report worse outcomes and their response to conventional and novel therapies.

## Data Availability

The data that support the findings of this study are available from RUDY but restrictions apply to the availability of these data, which were used under license for the current study, and so are not publicly available. Data are however available from the authors upon reasonable request and with permission of RUDY.

## Appendix

See Figs. 4, 5 and Tables 6, 7, 8, 9, 10, 11.

**Table 6:** Instrument rating scales

Health-related quality of life	Lowest level of quality of life	Highest level of quality of life
EQ-5D-5L	− 0.285	1
EQ-5D-3L (crosswalk)	− 0.594	1
SF-36-PCS	0	100
SF-36-MCS	0	100
*Sleep*	Highest level of sleep quality	Lowest level of sleep quality
PSQI	0	21
ESS	0	24
Lower normal daytime sleepiness	0	5
Higher normal daytime sleepiness	6	10
Mild excessive daytime sleepiness	11	12
Moderate excessive daytime sleepiness	13	15
Severe excessive daytime sleepiness	16	24
*Fatigue*	Highest level of fatigue	Lowest level of the fatigue
FACIT-F	0	160
Physical well-being	0	28
Social well-being	0	28
Emotional well-being	0	24
Functional well-being	0	28
Fatigue subscale	0	52
	Lowest level of the fatigue	Highest level of fatigue
FSS	9	63
*Pain*	Lowest level of pain	Highest level of pain
SF-MPQ-2	0	10
PainDETECT	− 1	38
Negative	− 1	12
Unclear	13	18
Positive	19	38
*Mental well-being*	Lowest level of anxiety/depression	Highest level of anxiety/depression
HADS-anxiety	0	21
Normal	0	7
Mild	8	10
Moderate/severe	11	21
HADS-depression	0	21
Normal	0	7
Mild	8	10
Moderate/severe	11	21

EQ-5D-5L (3L), EuroQol 5 dimensions-5 levels (3 levels); SF-36, Short-form 36; PCS, Physical Component Score; MCS, Mental Component Score; FSS, Fatigue Severity Scale; ESS, Epworth Sleepiness scale; PSQI, Pittsburgh sleep quality index; FACIT-F, Functional assessment of chronic illness therapy-fatigue; SF-MPQ-2, Short form McGill pain questionnaire version 2; HADS, Hospital anxiety and depression scale

**Table 7:** Cross-sectional summary statistics, by age group

Overall instrument score	Total sample	Age: < 40 years				Age: 40–60 years				Age: > 60 years				ANOVA *P*-value
	Range of submission dates	n	Mean	SD	Median	n	Mean	SD	Median	n	Mean	SD	Median	
*Health related quality of life*														
EQ-5D-5L (VT) Index score	07/2014–08/2019	19	0.643	0.294	0.728	18	0.708	0.220	0.760	11	0.570	0.302	0.649	0.411
EQ5D VAS score	07/2014–08/2019	19	63.42	26.58	74.00	18	67.22	23.17	68.00	11	53.09	28.10	55.00	0.358
EQ5D-3L (CW) Index score	07/2014–08/2019	19	0.558	0.326	0.664	18	0.604	0.241	0.663	11	0.465	0.345	0.567	0.490
SF-36- physical component score	07/2014–08/2019	18	35.55	15.10	35.88	16	32.01	14.77	28.86	10	28.55	11.46	28.94	0.455
SF-36- mental component score	07/2014–08/2019	18	47.77	9.94	49.28	16	46.23	11.74	46.64	10	52.91	8.39	54.26	0.273
*Fatigue*														
FSS score	09/2018–08/2019	7	28.57	11.30	25.00	10	34.60	17.16	32.50	6	34.83	12.12	33.50	0.651
FACIT-F score	04/2015–08/2019	18	104.33	30.51	102.33	16	100.92	28.37	108.00	9	110.67	30.90	108.00	0.737
FACIT-F- physical well-being	04/2015–08/2019	18	18.67	6.89	20.00	16	18.31	6.52	20.50	10	18.67	5.40	20.00	0.986
FACIT-F- social well-being	04/2015–08/2019	18	21.22	4.81	23.00	16	19.10	5.07	19.50	10	21.78	5.83	22.17	0.346
FACIT-F- emotional well-being	04/2015–08/2019	18	15.06	4.78	14.50	16	15.81	4.69	16.00	10	17.22	4.50	17.00	0.454
FACIT-F- functional well-being	04/2015–08/2019	18	18.33	5.24	17.50	16	17.63	5.14	17.50	10	18.89	6.84	19.00	0.806
FACIT-F- fatigue subscale	04/2015–08/2019	18	31.06	13.99	32.50	16	30.06	13.37	34.00	9	34.11	12.77	33.00	0.769
*Pain*														
SF-MPQ-2 score	07/2014–08/2019	19	1.89	1.55	1.91	16	2.01	1.72	1.50	11	1.89	1.25	1.86	0.968
Pain detect score	07–2014-08/2019	18	7.89	5.14	8.00	15	8.67	7.17	7.00	10	12.70	5.93	15.50	0.133
*Sleep*														
ESS score	09/2018–08/2019	7	5.00	3.00	5.00	10	5.40	3.17	6.00	6	7.67	5.05	7.00	0.389
PSQI score	07/2014–06/2019	18	8.11	5.05	7.00	14	9.64	3.56	10.50	9	9.22	2.77	9.00	0.570
*Mental wellbeing*														
HADS anxiety score	04/2015–06/2019	16	6.38	4.56	6.50	15	6.00	3.91	5.00	10	6.10	2.56	6.00	0.963
HADS depression score	04/2015–06/2019	16	4.31	3.52	4.50	15	4.73	3.13	4.00	10	5.30	4.19	4.50	0.789

The sample is the first submitted instrument from each participant

EQ-5D-5L (3L), EuroQol 5 dimensions-5 levels (3 levels); VAS, Visual analogue scale; SF-36, Short-form 36; PCS, Physical Component Score; MCS, Mental Component Score; FSS, Fatigue Severity Scale; ESS, Epworth Sleepiness scale; PSQI, Pittsburgh sleep quality index; FACIT-F, Functional assessment of chronic illness therapy-fatigue; SF-MPQ-2, Short form McGill pain questionnaire version 2; HADS, Hospital anxiety and depression scale

**Table 8:** Cross-sectional summary statistics for SF-36 scales, by sex

SF-36 dimensions	Total (n = 44)			Female (n = 35)			Male (n = 9)			T-test *P*-value
	Mean	SD	Median	Mean	SD	Median	Mean	SD	Median	
*Physical dimensions*										
Physical functioning	44.89	29.99	45.00	42.43	26.77	45.00	54.44	40.73	50.00	0.421
Physical role limitations	34.66	42.19	12.50	31.43	39.92	0.00	47.22	50.69	25.00	0.404
Pain	53.41	32.17	50.00	51.43	29.04	50.00	61.11	43.50	75.00	0.541
General health	44.20	23.72	40.00	41.23	22.15	40.00	55.78	27.39	67.00	0.169
*Mental dimensions*										
Emotional well-being	69.73	17.75	74.00	69.83	17.71	72.00	69.33	18.97	80.00	0.945
Emotional role limitations	65.91	40.34	83.33	63.81	42.30	100.00	74.07	32.39	66.67	0.440
Energy/fatigue	39.20	25.06	35.00	36.14	23.70	35.00	51.11	28.04	55.00	0.169
Social functioning	62.22	30.91	75.00	64.29	28.14	75.00	54.17	40.98	62.50	0.500

The sample is the first submitted instrument from each participant. The questionnaires were submitted between 07/2014 and 08/2019

SF-36, Short-form 36; PCS, Physical Component Score; MCS, Mental Component Score

**Table 9:** Cross-sectional summary statistics for SF-36 scales, by age

SF-36 dimensions	Total (n = 44)			Age: < 40 years (n = 18)			Age: 40–60 years (n = 16)			Age: > 60 years (n = 10)			ANOVA *P*-value
	Mean	SD	Median	Mean	SD	Median	Mean	SD	Median	Mean	SD	Median	
*Physical dimensions*													
Physical functioning	44.89	29.99	45.00	52.22	30.55	50.00	45.00	29.50	37.50	31.50	27.89	32.50	0.219
Physical role limitations	34.66	42.19	12.50	41.67	45.37	25.00	31.25	40.31	12.50	27.50	41.58	0.00	0.652
Pain	53.41	32.17	50.00	58.33	33.21	62.50	45.31	33.19	50.00	57.50	28.99	50.00	0.460
General health	44.20	23.72	40.00	46.00	28.12	44.50	44.00	21.81	42.50	41.30	19.68	35.00	0.886
*Mental dimensions*													
Emotional well-being	69.73	17.75	74.00	67.11	20.14	70.00	68.75	17.66	66.00	76.00	12.65	78.00	0.440
Emotional role limitations	65.91	40.34	83.33	72.22	36.60	100.00	54.17	41.94	66.67	73.33	43.89	100.00	0.352
Energy/fatigue	39.20	25.06	35.00	37.22	27.07	27.50	36.88	25.36	32.50	46.50	21.61	47.50	0.588
Social functioning	62.22	30.91	75.00	68.06	28.83	75.00	57.81	32.87	62.50	58.75	32.83	68.75	0.590

The sample is the first submitted instrument from each participant. The questionnaires were submitted between 07/2014 and 08/2019

SF-36, Short-form 36; PCS, Physical Component Score; MCS, Mental Component Score

**Table 10:** Cross-sectional summary statistics, by age and PROMs category

Instrument	Total		< 40 years		40–60 years		> 60 years	
	n	(%)	n	(%)	n	(%)	n	(%)
*ESS categories*								
Lower normal daytime sleepiness	12	(52.17%)	5	(71.43%)	5	(50.00%)	2	(33.33%)
Higher normal daytime Sleepiness	9	(39.13%)	1	(14.29%)	5	(50.00%)	3	(50.00%)
Mild excessive daytime sleepiness	1	(4.35%)	1	(14.29%)	0	(0.00%)	0	(0.00%)
Moderate excessive daytime sleepiness	0	(0.00%)	0	(0.00%)	0	(0.00%)	0	(0.00%)
Severe excessive daytime sleepiness	1	(4.35%)	0	(0.00%)	0	(0.00%)	1	(16.67%)
*Pain detect categories*								
Negative	28	(65.12%)	15	(83.33%)	10	(66.67%)	3	(30.00%)
Unclear	12	(27.91%)	3	(16.67%)	3	(20.00%)	6	(60.00%)
Positive	3	(6.98%)	0	(0.00%)	2	(13.33%)	1	(10.00%)
*HADS categories*								
*Anxiety*								
Normal	26	(63.41%)	9	(56.25%)	9	(60.00%)	8	(80.00%)
Mild	10	(24.39%)	4	(25.00%)	4	(26.67%)	2	(20.00%)
Moderate/severe	5	(12.20%)	3	(18.75%)	2	(13.33%)	0	(0.00%)
*Depression*								
Normal	35	(85.37%)	14	(87.50%)	13	(86.67%)	8	(80.00%)
Mild	2	(4.88%)	1	(6.25%)	1	(6.67%)	0	(0.00%)
Moderate/severe	4	(9.76%)	1	(6.25%)	1	(6.67%)	2	(20.00%)

ESS, Epworth Sleepiness scale; HADS, Hospital anxiety and depression scale

**Table 11:** Cluster sensitivity analysis, mean instrument scores

Instrument	Cluster 1 (n = 24) “Higher-score” (mean)	Cluster 2 (n = 9) “Lower-score” (mean)
EQ-5D-5L	0.782	0.281
PSQI	7.71	12.89
SF-36-PCS	38.35	18.38
SF-36-MCS	51.79	43.79
SF-MPQ-2	1.29	3.60
painDETECT	6.79	15.89
Negative, n (%)	20 (83.33)	2 (22.22)
Unclear, n (%)	4 (16.67)	4 (44.44)
Positive, n (%)	0 (0.00)	3 (33.33)

EQ-5D-5L, EuroQol 5 dimensions-5 levels; SF-36, Short-form 36; PCS, Physical Component Score; MCS, Mental Component Score; PSQI, Pittsburgh sleep quality index; SF-MPQ-2; Short form McGill pain questionnaire version 2

## Abbreviations

XLH: X-linked hypophosphataemia
PROMs: Patient reported outcome measures
HRQL: Health-related quality of life
EQ-5D-5L (3L): EuroQol 5 dimensions-5 levels (3 levels)
VAS: Visual analogue scale
SF-36: Short-form 36
PCS: Physical Component Score
MCS: Mental Component Score
FSS: Fatigue Severity Scale
ESS: Epworth Sleepiness scale
PSQI: Pittsburgh sleep quality index
FACIT-F: Functional assessment of chronic illness therapy-fatigue
SF-MPQ-2: Short form McGill pain questionnaire version 2
HADS: Hospital anxiety and depression scale
